# Procognitive, Anxiolytic, and Antidepressant-like Properties of Hyperoside and Protocatechuic Acid Corresponding with the Increase in Serum Serotonin Level after Prolonged Treatment in Mice

**DOI:** 10.3390/ph16121691

**Published:** 2023-12-05

**Authors:** Jolanta Orzelska-Górka, Katarzyna Dos Santos Szewczyk, Monika Gawrońska-Grzywacz, Mariola Herbet, Anna Lesniak, Anna Bielenica, Magdalena Bujalska-Zadrożny, Grażyna Biała

**Affiliations:** 1 Chair and Department of Pharmacology and Pharmacodynamics, Medical University of Lublin, Chodzki 4A, 20-093 Lublin, Poland; grazyna.biala@umlub.pl; 2 Department of Pharmaceutical Botany, Medical University of Lublin, Chodzki 1, 20-093 Lublin, Poland; katarzyna.szewczyk@umlub.pl; 3 Chair and Department of Toxicology, Faculty of Pharmacy, Medical University of Lublin, Jaczewskiego 8b, 20-090 Lublin, Poland; monika.gawronska-grzywacz@umlub.pl (M.G.-G.); mariola.herbet@umlub.pl (M.H.); 4 Department of Pharmacotherapy and Pharmaceutical Care, Faculty of Pharmacy, Medical University of Warsaw, Centre for Preclinical Research and Technology, 02-097 Warsaw, Poland; alesniak.imdik@gmail.com (A.L.); magdalena.bujalska@wum.edu.pl (M.B.-Z.); 5 Department of Biochemistry, Medical University of Warsaw, 02-097 Warsaw, Poland; anna.bielenica@wum.edu.pl

**Keywords:** hyperoside, protocatechuic acid, antidepressant, anxiolytic, procognitive, mice

## Abstract

Two polyphenols–hyperoside (HYP) and protocatechuic acid (PCA) were reported to exert antidepressant activity in rodents after acute treatment. Our previous study also showed that this activity might have been influenced by the monoaminergic system and the upregulation of the brain-derived neurotropic factor (BDNF) level. A very long-term pharmacological therapy is required for the treatment of a patient with depression. The repetitive use of antidepressants is recognized to impact the brain structures responsible for regulating both emotional and cognitive behaviors. Thus, we investigated the antidepressant, anxiolytic, and procognitive effects of HYP and PCA in mice after acute and prolonged treatment (14 days). Both polyphenols induced an anxiogenic-like effect after acute treatment, whereas an anxiolytic effect occurred after repetitive administration. PCA and HYP showed procognitive effects when they were administered acutely and chronically, but it seems that their influence on long-term memory was stronger than on short-term memory. In addition, the preset study showed that the dose of 7.5 mg/kg of PCA and HYP was effective in counteracting the effects of co-administered scopolamine in the long-term memory impairment model induced by scopolamine. Our experiments revealed the compounds have no affinity for 5-HT_1A_ and 5-HT_2A_ receptors, whereas a significant increase in serum serotonin level after prolonged administration of PCA and HYP at a dose of 3.75 mg/kg was observed. Thus, it supports the involvement of the serotonergic system in the polyphenol mechanisms. These findings led us to hypothesize that the polyphenols isolated from *Impatiens glandulifera* can hold promise in treating mental disorders with cognitive dysfunction. Consequently, extended studies are necessary to delve into their pharmacological profile.

## 1. Introduction

Mental disorders likely comprise multiple disturbances with overlapping symptoms and diverse etiologies. This causes great diagnostic and therapeutic difficulties. Thus, antidepressants are presently given as extended prophylactic treatment post remission of depressive symptoms, and their prescription extends to various other psychiatric and neurological conditions apart from major depressive disorder (MDD). MDD stands as one of the most widespread and extensively comorbid psychiatric conditions that cause severe disability. It is characterized by complex etiology because it can manifest as a gradual loss of enjoyment of life, anxiety, depressive thoughts, sleep disturbances, and impairments in cognition and motivation [1]. It should be underlined that, according to several authors, cognitive impairments are a core component of MDD [2]. MDD is often a long-term condition, occurring with relapses and recurrences, which tends to become a chronic disease. Unfortunately, the increasing impact of MDD is not paralleled by improvements in the understanding of its etiology and its treatment. During the 1960s, the monoaminergic hypothesis emerged, suggesting that a reduction in various neurotransmitters, like serotonin (5-HT, 5-hydroxytryptamine), dopamine (DA), and noradrenaline (NE), within the central nervous system (CNS) contributes to the onset of depression. This hypothesis finds support in the mechanism of action of antidepressant medications, which elevate monoamine levels and alleviate symptoms of depression [3]. Thus, the discovery and dissemination of first-generation medications (monoamine oxidase inhibitors (MAOIs) and tricyclic antidepressants (TCAs)) initially revolutionized the treatment of depression. However, this treatment is responsible for severe side effects that forced many patients to abandon pharmacotherapy. Typical side effects of currently available antidepressants include tolerance, the recurrence of disease, sexual dysfunction, cognitive impairment, and weight gain [4]. Second-generation antidepressants (serotonin-norepinephrine reuptake inhibitors [SNRIs] and selective serotonin reuptake inhibitors (SSRIs), among others) are superior to first-generation antidepressants in terms of side effects but not in overall efficacy. In addition, these compounds (the first and the second generation) often take weeks or months to reach full effectiveness [5]. Consequently, there is still a need for the search for a well-tolerated and effective therapy for MDD.

**Figure 1:**
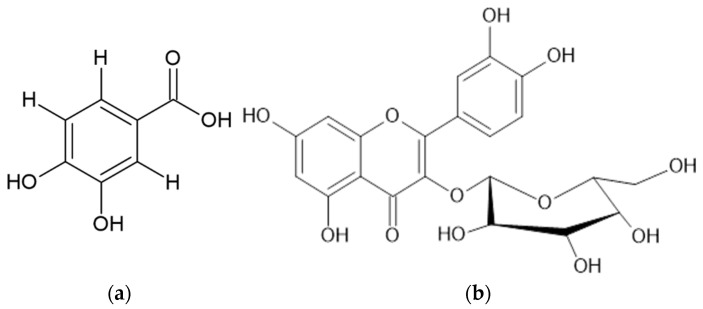
Structures of protocatechuic acid (**a**) and hyperoside (**b**).

Natural products have garnered escalating interest in preventing and treating psychiatric disorders, notably depression. Numerous natural products exhibit comparable effectiveness to prescription medications while often presenting little to no side effects [6]. Polyphenolic compounds, such as flavonoids and phenolic acids, are known to have a wide repertoire of biological properties, especially because of their impact on the CNS, including their potential antidepressant-like effects [3]. Antidepressant activities of polyphenolic compounds have been related to the increase in the bioamine content because of the restriction of the 5-HT, DA, and NE reuptake by synaptosomes and MAO activities [7]. *Impatiens glandulifera* Royle, commonly known as Himalayan Balsam and belonging to the Balsaminaceae family, is an annual invasive species in Europe. Phytochemical investigations have revealed the presence of biologically active compounds within this species, including flavonoids [8,9,10] and phenolic acids [11,12]. Some of these compounds have demonstrated antidepressant effects [13,14]. Our recent findings showed that hydroethanolic extracts of *I. glandulifera* possess anxiolytic effects in the elevated plus-maze test (EPM) conducted on mice [8]. In our previous studies, we also focused on the investigation of acute antidepressant properties of two polyphenols– protocatechuic acid (PCA; Figure 1a) and hyperoside (HYP; Figure 1b) isolated from *Impatiens glandulifera*. Our results demonstrated that HYP and PCA induce antidepressant-like effects in mice following acute treatment, potentially mediated by the monoaminergic system and the elevation of BDNF levels [15]. It should be noted that there are data confirming the penetration of PCA and HYP into the CNS. PCA—as a low molecular compound—can easily cross the blood–brain barrier (BBB) (for review, see [16]). It was shown that the oral intake of PCA increased PCA concentration in plasma and organs such as the heart, brain, liver kidneys, and lungs in mice. The main metabolite of HYP, miquelianin, was demonstrated to cross all barriers and enter the brain after oral administration (for review, see [17]).

As a continuation of our previous experiments, the present study had four objectives. We aimed (1) to establish the anxiolytic and procognitive-like effect of PCA and HYP in acute treatment, (2) to establish whether antidepressant-, anxiolytic-, and procognitive-like effects of the polyphenols could be maintained in the long term by repeated daily treatment, and (3) to determine whether chronic administration elevated peripheral serotonin levels. Finally, we aimed (4) to investigate whether acute treatment with HYP and PCA would prevent the memory impairment induced by scopolamine (a typical anticholinergic drug).

## 2. Results

### 2.1. Affinities of PCA and HYP at 5-HT_1A_ and 5-HT_2A_ Receptors

According to Table 1, the affinities of both HYP and PCA at the 5-HT_1A_ and 5-HT_2A_ receptors fell within the micromolar range. In addition, 5-HT_1A_ and 5-HT_2A_ receptor binding affinities obtained for HYP and PCA were over three and five orders of magnitude lower when compared to 5-HT and ketanserin, respectively (*p* > 0.05). Both HYP and PCA exhibited similar affinities at the 5-HT_1A_ receptor—42.9 µM vs. 33.3 µM, respectively (F_(1,69)_ = 1.3, *p* = 0.22). The same trend was also observed for their 5-HT_2A_ receptor affinities (F_(1,69)_ = 0.2, *p* = 0.63). Of note, HYP and PCA also showed low selectivity and were 9.18 and 6.15 times more selective, respectively, at the 5-HT_1A_ receptor.

### 2.2. Effect of Acute PCA or HYP Administration on the Mice Behavior Assessed in the EPM

**Figure 2:**
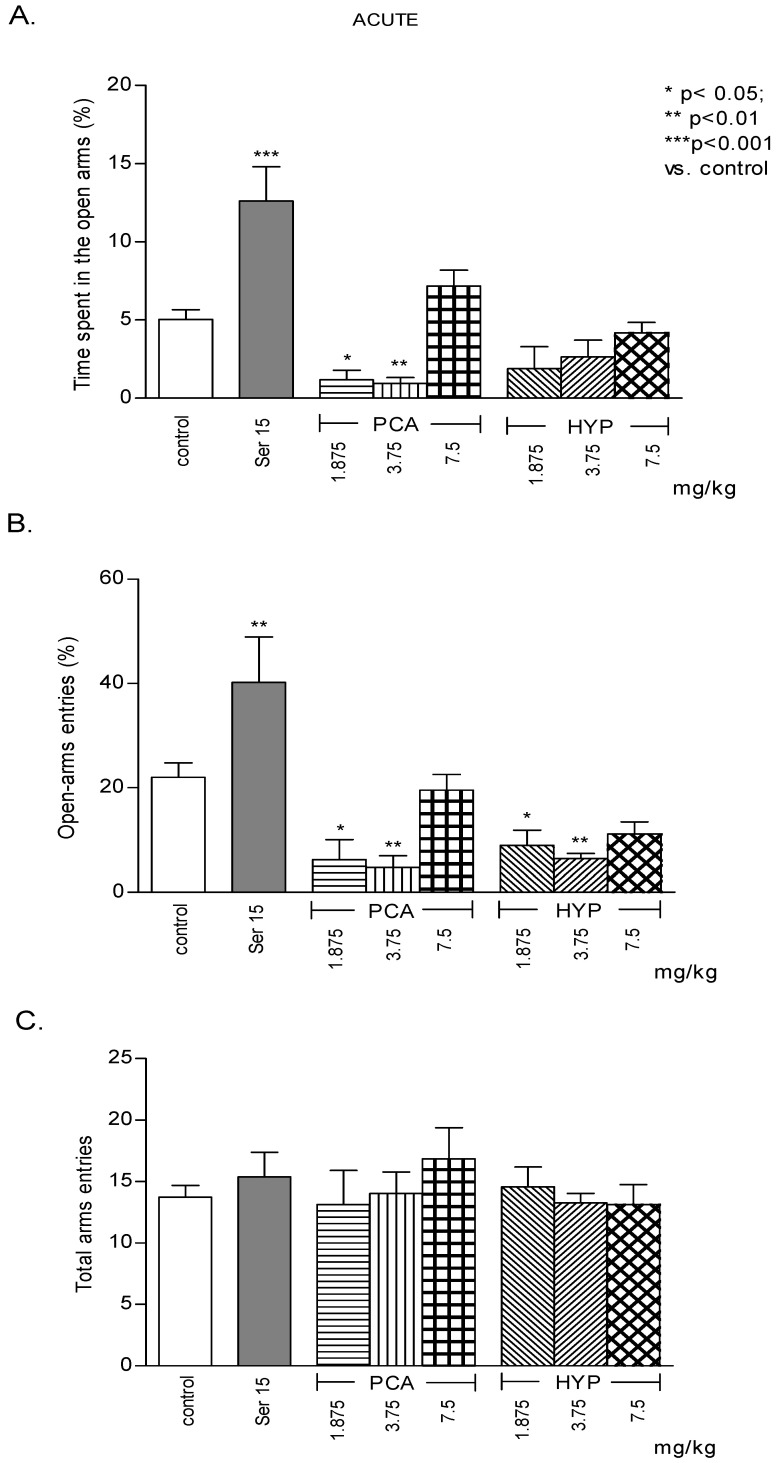
The influence of the investigated compounds PCA, HYP, and Ser, administered acutely, on elevated plus-maze (EPM) performance in mice. Percentage of time spent in open arms (**A**), the percentage of the open-arm entries (**B**), and total arm entries (**C**). PCA, HYP (1.875, 3.75, and 7.5 mg/kg), and Ser (15 mg/kg) were administered i.p. 60 min before the test. The results are expressed as mean ± SEM; *** *p* <0.001; ** *p* < 0.01; * *p* < 0.05 vs. control (Bonferroni’s test).

The analysis using one-way ANOVA revealed notable alterations in the percentage of time spent in open arms and the percentage of entries into open arms following the administration of Ser, PCA, and HYP (F_(7,60)_ = 15.11, *p* < 0.0001, Figure 2A and F_(7,63)_ = 10.68, *p* < 0.0001, Figure 2B, respectively). However, there were no significant changes observed in total arm entries (Figure 2C). A subsequent Bonferroni’s post hoc test affirmed a considerable increase in the time spent in open arms and the percentage of entries into open arms after Ser administration at a dosage of 15 mg/kg. Conversely, PCA (1.875 and 3.75 mg/kg) and HYP (1.875 and 3.75 mg/kg) notably reduced the percentage of time spent in open arms and the percentage of open-arm entries (*p* < 0.05 and *p* < 0.01, respectively).

### 2.3. Effect of Chronic (14 Days) PCA or HYP Administration on Mice Behavior Assessed in the EPM

**Figure 3:**
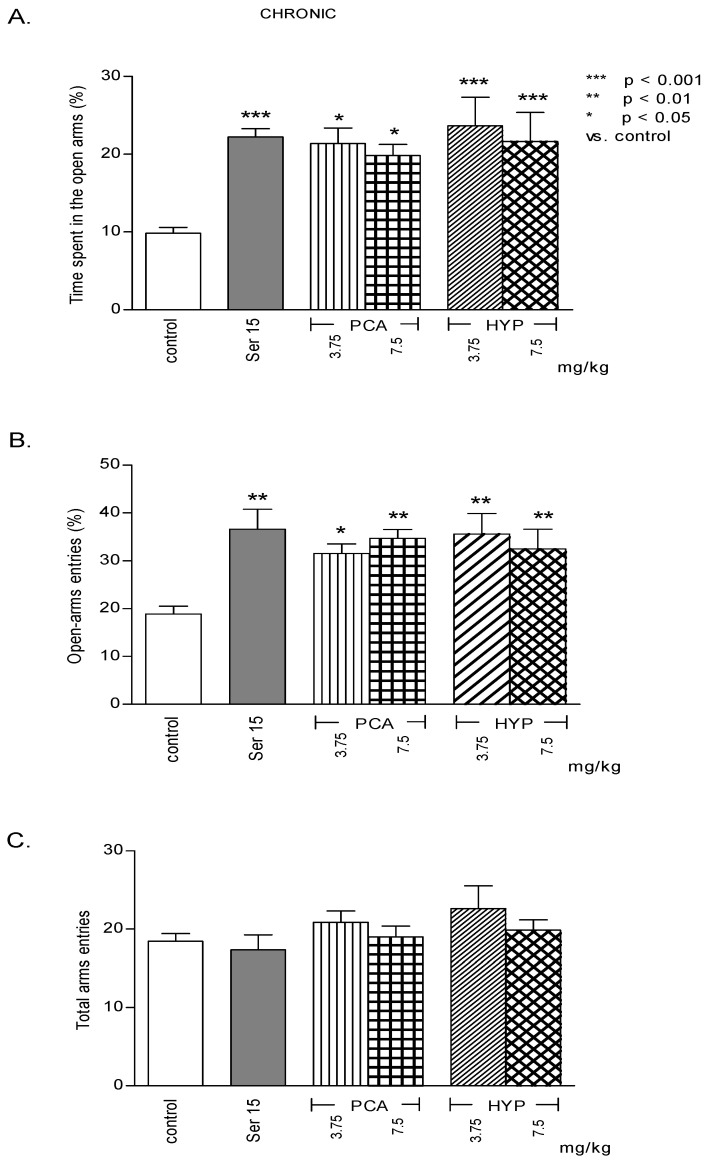
The chronic administration of the investigated compounds PCA, HYP, and Ser and their impact on mice performance in the elevated plus-maze (EPM), as represented by the percentage of time spent in open arms (**A**), the percentage of open-arm entries (**B**), and total arm entries (**C**). PCA, HYP (3.75 and 7.5 mg/kg), and Ser (15 mg/kg) were intraperitoneally administered for 14 consecutive days. The EPM was conducted three days following the final injection. Results are presented as mean ± SEM; *** *p* < 0.001; ** *p* < 0.01; * *p* < 0.05 compared to the control group (Bonferroni’s test).

One-way ANOVA revealed that the repeated administration of PCA or HYP at doses 3.75 and 7.5 mg/kg and Ser (15 mg/kg) exerted a statistically significant effect on the time spent in open arms and on the open-arm entries (F_(5,48)_ = 5.942; *p* = 0.002; Figure 3A and F_(5,47)_ = 4.216; *p* = 0.003; Figure 3B). The applied post hoc Bonferroni’s test showed PCA (3.75 and 7.5 mg/kg), HYP (3.75 and 7.5 mg/kg), and Ser (15 mg/kg) to have significantly increased the time spent in the open arms (*p* < 0.05 and *p* < 0.05; *p* < 0.001 and *p* < 0.001; *p* < 0.001, respectively) and to have significantly increased in the open-arm entries (*p* < 0.05 and *p* < 0.01; *p* < 0.01 and *p* < 0.01; *p* < 0.01, respectively), as compared with the saline-treated control group, indicating that these constituents and Ser exert anxiolytic-like effects.

### 2.4. Effect of Chronic (14 Days) PCA or HYP Administration on the Mice Behavior Assessed in the FST

**Figure 4:**
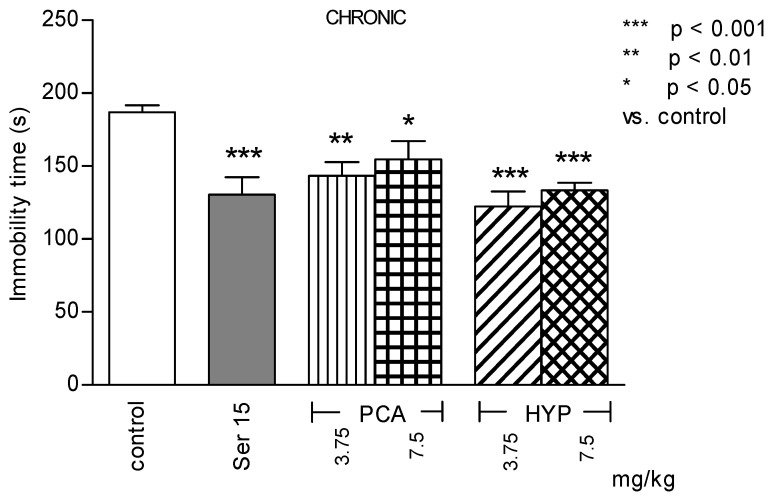
The influence of studied compounds PCA, HYP, and Ser, administered chronically, on the immobility time in the FST. PCA, HYP (3.75 and 7.5 mg/kg), and Ser (15 mg/kg) were administered i.p. for 14 consecutive days. Three days after the last injection, the FST was performed. The results are expressed as mean ± SEM; *** *p* < 0.001; ** *p* < 0.01; * *p* < 0.05 vs. control (Bonferroni’s test).

One-way ANOVA revealed a statistically significant effect of the chronic intraperitoneal doses of PCA (3.75 and 7.5 mg/kg), HYP (3.75 and 7.5 mg/kg), and Ser (15 mg/kg) on immobility time values (F_(5,48)_ = 7.049; *p* < 0.0001; Figure 4). A subsequent Bonferroni’s post hoc test indicated that PCA (3.75 and 7.5 mg/kg), HYP (3.75 and 7.5 mg/kg), and Ser (15 mg/kg) significantly reduced immobility time in comparison to the saline-treated control group (*p* < 0.01 and *p* < 0.05; *p* < 0.001 and *p* < 0.001; and *p* < 0.001, respectively). These findings suggest an antidepressant-like effect associated with these compounds and Ser.

### 2.5. Effects of Acute or Chronic PCA or HYP Administration on the Short-Term Memory Acquisition of Mice Assessed in the NOR

**Figure 5:**
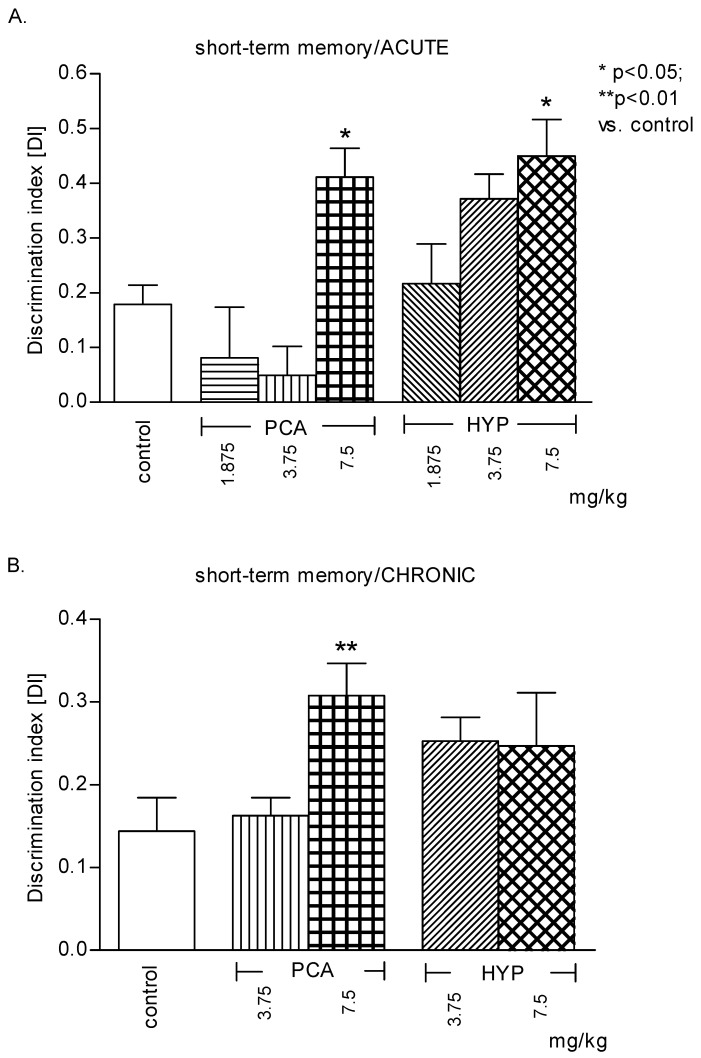
The influence of acute (**A**) or chronic (**B**) PCA or HYP administration on memory-related responses, expressed as discrimination index (DI) during the short-term acquisition trial, using NOR test in mice. In the acute experiment, PCA and HYP (1.875, 3.75, and 7.5 mg/kg) were administered i.p. 60 min before the first trial (T1) of the NOR task. Short-term memory performance was assessed 1 h after the first trial. In the chronic experiment, PCA and HYP (3.75 and 7.5 mg/kg) were administered i.p. for 14 consecutive days. Short-term memory performance was assessed 24 h after the last injection. The results are expressed as mean ± SEM; ** *p* < 0.01; * *p* < 0.05 vs. control (Bonferroni’s test).

One-way ANOVA revealed statistically significant effects of acute i.p. doses of PCA (7.5 mg/kg) and HYP (7.5 mg/kg) on DI values (F_(6,46)_ = 6.576; *p* < 0.001) (Figure 5). The applied post hoc Bonferroni’s test revealed a significant increase of DI by PCA (7.5 mg/kg) and HYP (7.5 mg/kg), as compared with the saline-treated control group (*p* < 0.05), confirming that PCA and HYP improved short-term memory processes after acute treatment (see Figure 5A), while lower doses of PCA and HYP (1.875 and 3.75 mg/kg) did not affect DI.

One-way ANOVA identified statistically significant effects of chronic intraperitoneal doses of PCA (7.5 mg/kg) on DI values (F(4,35) = 3.298; *p* = 0.0215). Bonferroni’s post hoc test further demonstrated a significant increase in DI by PCA (7.5 mg/kg) compared to the saline-treated control group (*p* < 0.01). This confirms the improvement of short-term memory processes due to repeated administration of PCA (as depicted in Figure 5B), while the lower dose of PCA (3.75 mg/kg) and the used doses of HYP (3.75 and 7.5 mg/kg) did not affect DI.

As shown in Table 2 in this set of experiments, data analysis indicated that PCA (1.875, 3.75, and 7.5 mg/kg) and HYP (1.875 and 3.75 mg/kg) did not change total exploration time in T2, whereas the acute administration of HYP at a dose of 7.5 mg/kg showed a statistically significant increase in the total exploration of objects in T2 compared to saline-treated group (*p* < 0.01, one-way ANOVA, post hoc Bonferroni’s test).

### 2.6. Effects of Acute or Chronic PCA or HYP Administration on the Long-Term Memory Acquisition of Mice Assessed in the NOR

**Figure 6:**
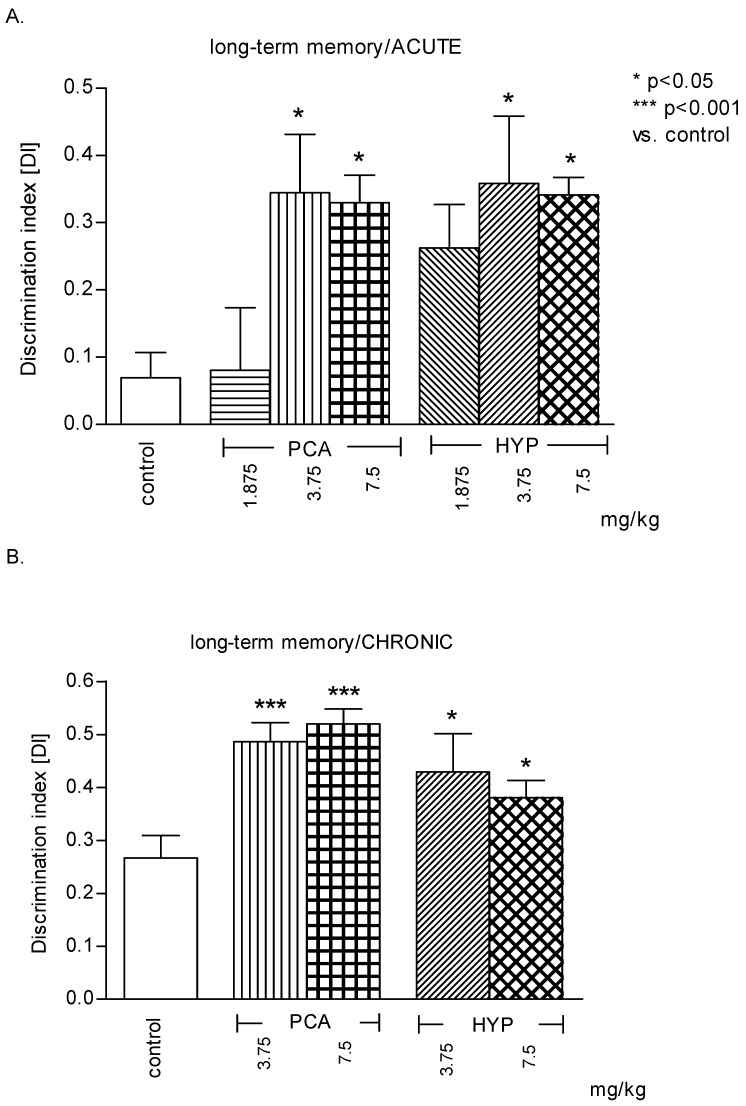
The influence of acute (**A**) or chronic (**B**) PCA or HYP administration on the memory-related responses, expressed as discrimination index (DI) during the long-term acquisition trial, using NOR test in mice. In the acute experiment, PCA and HYP (1.875, 3.75, and 7.5 mg/kg) were administered i.p. 60 min before the first trial (T1) of the NOR task. Long-term memory performance was assessed 24 h after the first trial. In the chronic experiment, PCA and HYP (3.75 and 7.5 mg/kg) were administered i.p. for 14 consecutive days. Long-term memory performance was assessed 48 h after the last injection. The results are presented as mean ± SEM; *** *p* < 0.001; * *p* < 0.05 vs. control (Bonferroni’s test).

One-way ANOVA revealed statistically significant effects of acute i.p. doses of PCA (3.75 and 7.5 mg/kg) and HYP (3.75 and 7.5 mg/kg) on DI values (F_(6,54)_ = 4.048; *p* = 0.002). The applied post hoc Bonferroni’s test revealed a significant increase of DI by both compounds—PCA and HYP—at two used doses (3.75 and 7.5 mg/kg), as compared with the saline-treated control group (*p* < 0.05), confirming that PCA and HYP improved long-term memory processes after an acute treatment (see Figure 6A).

One-way ANOVA revealed statistically significant effects of chronic i.p. doses of PCA (3.75 and 7.5 mg/kg) and HYP (3.75 and 7.5 mg/kg) on DI values (F_(4,35)_ = 8.594; *p* < 0.001). The applied post hoc Bonferroni’s test revealed a significant increase of DI by both compounds—PCA and HYP—at two used doses (3.75 and 7.5 mg/kg), as compared with the saline-treated control group (*p* < 0.001 for PCA and *p* < 0.05 for HYP), confirming that PCA and HYP improved long-term memory processes (see Figure 6B) after repeated administration.

As depicted in Table 3 from these experiments, the data analysis revealed that no doses of PCA and HYP altered the total exploration time in T3.

### 2.7. Effects of an Acute PCA or HYP Administration on the Scopolamine-Induced Short-Term (Figure 7A) or Long-Term (Figure 7B) Memory Impairment of Mice Assessed in the NOR

**Figure 7:**
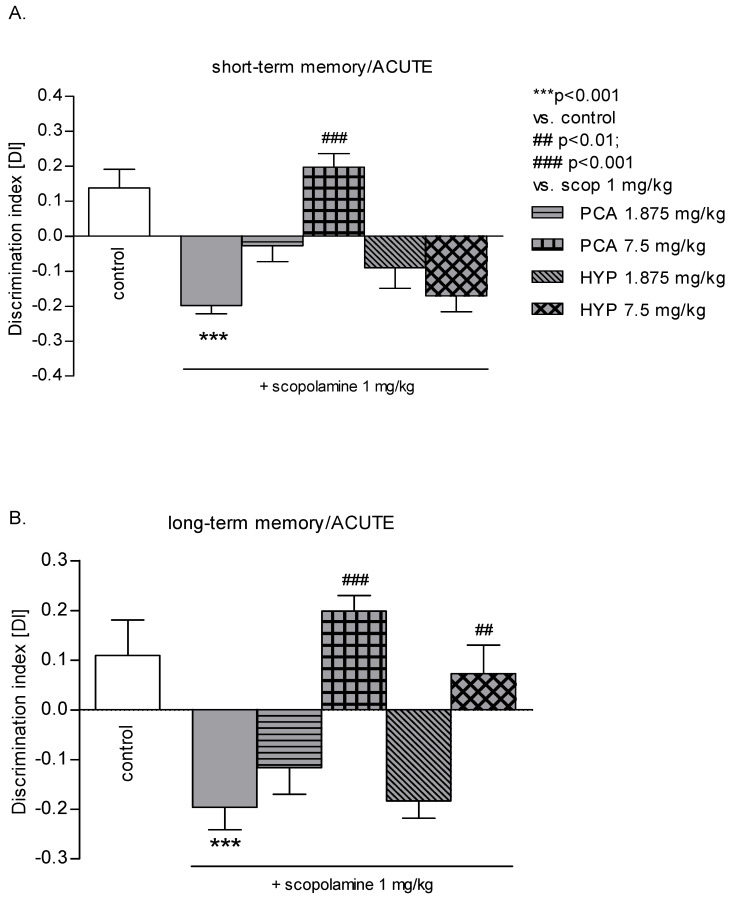
The influence of acute administration of PCA, HYP, and/or scopolamine on the memory-related responses, expressed as discrimination index (DI) during the short-(**A**) and long-term (**B**) acquisition trial, using NOR test in mice. HYP, PCA (1.875 and 7.5 mg/kg), or saline and/or scopolamine (1 mg/kg) were administered i.p. to mice 60 min and/or 15 min (for scopolamine) before the acquisition trial of NOR test. The compounds were injected combined with scopolamine. The results are presented as mean ± SEM; *** *p* < 0.001 vs. control; ## *p* < 0.01, ### *p* < 0.001 vs. scopolamine-treated group (Bonferroni’s test).

One-way ANOVA revealed statistically significant effects of acute i.p. doses of PCA (7.5 mg/kg), HYP (7.5 mg/kg), and/or scopolamine (1 mg/kg) on DI values (F_(5,47)_ = 12.89; *p* < 0.001) during the short-term memory acquisition in the NOR test. The subsequent Bonferroni’s post hoc test demonstrated a noteworthy reduction in DI caused by scopolamine (1 mg/kg) compared to the saline-treated mice (*p* < 0.001), confirming the amnesic effect of the drug. Remarkably, this effect was reversed by PCA administered at a dose of 7.5 mg/kg (*p* < 0.001) (refer to Figure 7A).

One-way ANOVA revealed statistically significant effects of acute i.p. doses of PCA (7.5 mg/kg), HYP (7.5 mg/kg), and/or scopolamine (1 mg/kg) on DI values (F_(5,48)_ = 12.11; *p* < 0.001) during the long-term memory acquisition in the NOR test. The applied post hoc Bonferroni’s test revealed a significant decrease of DI by scopolamine (1 mg/kg) in comparison with the saline-treated mice (*p* < 0.001), confirming the amnesic effect of the drug. This effect was reversed by PCA and HYP at doses of 7.5 mg/kg (*p* < 0.001 and *p* < 0.01, respectively) (see Figure 7B).

In this particular experiment, the analysis presented in Table 4 demonstrated that across all doses administered, both PCA and HYP did not induce any alterations in the total exploration time observed during T2 and T3.

### 2.8. Effect of Chronic (14 Days) PCA or HYP Administration on the Locomotor Activity of Mice

As shown in Table 5, data analysis indicated that no doses of PCA and HYP after repeated administration changed the distance traveled by mice.

### 2.9. Effect of PCA, HYP, and/or Scopolamine Acute Administration on the Locomotor Activity of Mice

One-way ANOVA revealed that an acute administration of PCA, HYP, and/or scopolamine had a statistically significant effect on mice locomotion, both within 6 min of measurement (F_(9,69)_ = 13.78, *p* < 0.0001) and within 60 min of measurement (F_(9,70)_ = 12.11, *p* < 0.0001). The applied post hoc Bonferroni’s test showed that scopolamine (1 mg/kg) substantially increased the distance traveled by mice measured within 6 min (*p* < 0.001) and 60 min (*p* < 0.01; Table 6).

### 2.10. Changes in Body Weight (Means ± SEM) in Mice Chronically Administered with Saline, Ser (15 mg/kg), PCA, or HYP (3.75 mg/kg)

**Figure 8:**
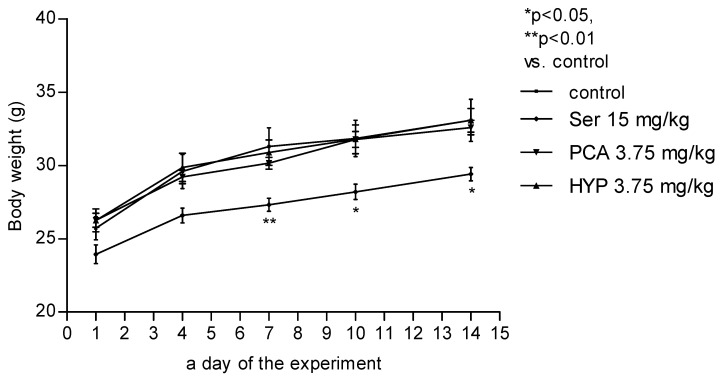
Changes in body weight (means ± SEM) in mice chronically administered with saline, Ser (15 mg/kg), PCA, or HYP (3.75 mg/kg). Body weight was monitored on the 1st, 4th, 7th, 10th, and 14th day of the chronic experiment. The results are expressed as mean ± SEM; ** *p* < 0.01; * *p* < 0.05 vs. control (Bonferroni’s test).

Two-way ANOVA revealed statistically significant effects of the treatment (F_(12,138)_= 0.25; *p* = 0.9950) and time (F_(4,138)_= 37.47; *p* < 0.0001). Bonferroni’s post hoc test revealed significantly less weight gain in Ser-treated animals compared to the control group on the 7th, 10th, and 14th day of the experiment (*p* < 0.01 and *p* < 0.05, respectively; Figure 8). 

### 2.11. The Influence on Serotonin Level in the Serum of Mice after Acute (A) and Chronic (B) Exposure of PCA, HYP, and Ser (Figure 9)

**Figure 9:**
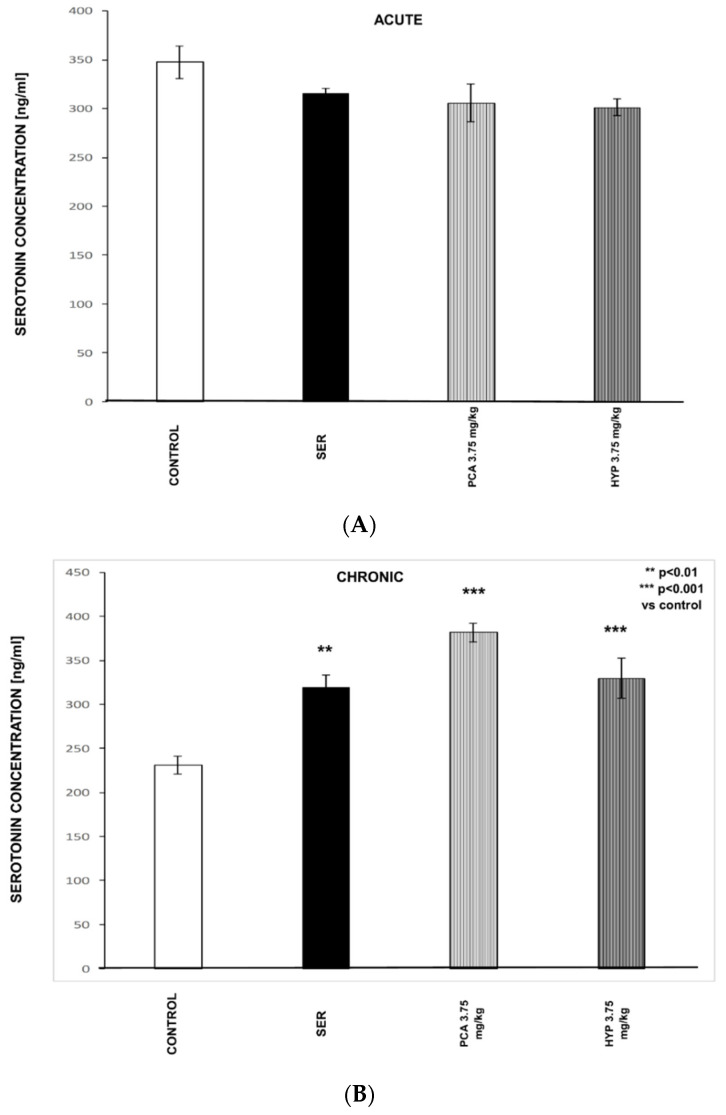
The influence on serotonin level in the serum of mice after acute (**A**) and chronic (**B**) exposure to PCA, HYP, and Ser. The compounds were administered i.p. to mice 60 min or 14 consecutive days before serum collection. Control group received 0.9% NaCl (saline).

In the serum of mice exposed to acute treatment with Ser or tested natural origin polyphenols (PCA or HYP), no significant changes in the concentration of serotonin in the serum were noted (*p* > 0.05; Figure 9A). However, chronic treatment with Ser led to a very significant enhancement of serum serotonin level (F_(3.05)_ = 18.631, *p* < 0.01; Figure 9B) in comparison to the control group. However, in the group of mice treated for 14 days with PCA or HYP at a dose of 3.75 mg/kg i.p., a notably substantial increase in serotonin concentration was observed compared to the control group (F[3.05] = 18.631, *p* < 0.001; Figure 9B).

## 3. Discussion

Regarding the four outlined objectives in the Introduction, the experiments conducted demonstrate that (1) PCA and HYP in the acute treatment exert anxiogenic-like and procognitive effects; (2) antidepressant and procognitive-like effects of both polyphenols are maintained in the long-term administration, whereas anxiolytic effect of the compounds appears after prolonged treatment; (3) chronic treatment with PCA and HYP elevates peripheral serotonin levels; and (4) reversal by PCA and HYP results in a dyscognitive effect of scopolamine.

Firstly, we checked the influence of two polyphenols—PCA and HYP—on the mice’s behavior in the EPM after a single administration. A significant anxiogenic-like effect was seen over a limited dose range (1.875–3.75 mg/kg) on conventional indices of anxiety, including a decreased percentage of open entries (for PCA) and/or open-arm time (for both polyphenols) [18]. This effect was lost at the highest used dose—7.5 mg/kg. One potential explanation is that the anxiogenic effect of PCA and HYP observed at only two lower doses after acute treatment was due to the limited dose ranges. It is known that the influence of some compounds (e.g., WAY-100635, a 5-HT_1A_ receptor antagonist) on anxiety-related behavior ranged from anxiolysis to anxiogenesis with an apparent bell-shaped dose–response relationship [19]. It can also be speculated that adaptive changes in the brain are needed for the anxiolytic effect to occur. This speculation can be confirmed by the result of the next experiment. Interestingly, after prolonged treatment (14 days of daily administration), an anxiolytic effect of both polyphenols at both used doses (3.75 and 7.5 mg/kg) was observed. PCA and HYP produced a behavioral profile indicative of anxiety reduction by the increase in the percentage of time spent in the open arms and open-arm entries. It should be underlined that these effects were not confounded by changes in locomotor activity, and they were observed equally at two used doses.

We confirmed that, in addition to their anxiolytic-like effect after chronic treatment, PCA and HYP also exerted an antidepressant effect in the FST. The decrease in immobility time in this paradigm reflects the antidepressant-like activity [20]. In the present study, we found that repeated daily administration of both polyphenols (3.75 and 7.5 mg/kg, i.p.) for 14 consecutive days significantly decreased the immobility time in the FST. These results indicate that the potential antidepressant-like effect of PCA and HYP is maintained in the long-term treatment. Importantly, the antidepressant efficacy of HYP in the FST was statistically higher at the same doses, i.e., 3.75 and 7.5 mg/kg, after prolonged treatment in comparison to the acute treatment. This is a common phenomenon for currently used antidepressants, e.g., SSRIs or SNRIs, as the antidepressant effect necessitates adaptive alterations at the neuronal receptor level. Likewise, fluoxetine [21], *Hypericum perforatum* [22], or a newly developed substance with potential antidepressant activity (a dual 5-HT_1A_ and 5-HT_7_ receptor antagonist, HBK-15) [23] demonstrated antidepressant-like effects with lower doses upon chronic administration compared to the doses effective after acute injection. It is important to note that the antidepressant-like effect of HYP after chronic treatment was comparable to Ser, a widely used antidepressant. Furthermore, another interesting observation arising from the above-outlined results was no evidence that tachyphylaxis/tolerance develops to the antidepressant-like activity of PCA and HYP upon repeated treatment. It is an important pharmacological feature of both polyphenols, considering that pharmacotherapy of depression in humans requires chronic administration of antidepressants [24,25]. Our above findings related to PCA are in partial disagreement with the study in which the olfactory bulbectomized (OBX) rat model was used. PCA after chronic treatment (14 days) was proposed to be able to attenuate OBX-induced depressive-like behaviors by improving behavioral and neurobiochemical alternations. But, in the sham groups, the antidepressant effect of PCA measured in the FST was not observed [26]. Our study related to the antidepressant features of HYP is in support of the results from a behavioral study on rats that revealed that HYP (0.6 mg/kg) administered orally for twelve days still significantly decreased immobility time in the FST [27].

Cognitive decline forms a significant aspect of the clinical presentation in individuals with depression, and it is included as one of the diagnostic criteria of depressive disorders in the DSM-5 classification [2]. Bearing that in mind, specifically, it is important to analyze the ability of the new compounds with potential antidepressant properties to improve affective symptoms as well as cognitive dysfunctions. In addition, systemic administration of the compounds can be responsible for their effects on different brain structures that are involved in the regulation of both emotional and cognitive behaviors. Consequently, in addition to their potential mood-enhancing effects, PCA and HYP can impact learning and memory [28]. Thus, the next experiment was focused on the putative procognitive activity of PCA and HYP assessed in the NOR test, followed by the determination of the antiamnestic properties of one effective and one ineffective dose. Unlike numerous cognitive tests, NOR does not incorporate rewards or punishments, ensuring behavioral responses remain uninfluenced by reinforcement/response dynamics. Successful recognition of an object is indicated by the animal spending comparatively more time investigating an unfamiliar object than one presented recently [29]. The NOR test facilitated the evaluation of both short-term and long-term memory, determined by the duration between the pre-test and the subsequent retention test [30]. Short-term memory was evaluated with a trial conducted one hour after the training session. Assessing long-term memory involved measuring latency time after 24 h. In both instances, drugs were administered before the pre-test, aiming to influence the natural acquisition of information [31]. The NOR test performed for both polyphenols clearly demonstrated that either short- or long-term memory was improved, depending upon the used polyphenols and their doses. Administering the highest dose of PCA and HYP (7.5 mg/kg) prior to the introductory session resulted in heightened interest in a novel object compared to a familiar one during the recognition session, indicating an enhancement in the memory acquisition stage. The above effects were observed 1 h after the introductory section, which showed the short-term memory-enhancing effects induced by polyphenols. At a dosage of 3.75 mg/kg, HYP showed a tendency toward enhancing cognition; however, the results obtained did not reach statistical significance. Importantly, it seems that the influence of PCA and HYP on long-term memory was stronger than on short-term memory. PCA and HYP showed memory-enhancing properties also at a lower used dose—3.75 mg/kg—when measured 24 h after the introductory session. The first conclusion is to suppose that this result could be due to an effect of the polyphenol during the introductory session, but also afterward, so an effect on consolidation [31]. The second hypothesis, which is conjectural, revolves around the potential activity of PCA and HYP, which might manifest over an extended duration [30]. Furthermore, daily treatment with PCA and HYP causes sustained short- and long-term procognitive effects. The memory-enhancing properties of PCA observed after repeated administration were significantly stronger than after a single injection, whereas the same activity of HYP was maintained at the same level. Conversely, the present findings contradict an earlier study by Krzysztoforska et al. [32], which showed that PCA did not produce a significant impact on memory performance in healthy rats. The above discrepancy might depend on differences in experimental settings, among which were the different doses of the compound, the different routes of administration, and the different behavioral paradigms used.

Another interesting observation arising from the present study was the fact that PCA and HYP displayed procognitive properties in the same doses as they showed anxiolytic and antidepressant effects concerning the chronic treatment. It is important to highlight that the administration of PCA and HYP at the current doses had no impact on locomotor activity, as evaluated by the distance traveled, nor did it affect the total exploration time. The observed patterns of results dismiss the possibility that the impact of polyphenols on the mice’s cognitive performance was associated with their level of movement or exploratory behavior. We observed one exception to what we wrote above, namely HYP at a dose of 7.5 mg/kg significantly increased the exploration time measured 60 min after the initial session, which might have affected the ability of mice to recognize objects in this phase. We cannot provide a plausible explanation for these effects since there was no change in the total exploration time in the other experiments. The object recognition task relies on spontaneous exploration, and as this behavior can be linked to either anxiogenic or anxiolytic processes, the compounds we administered could have influenced animal performance through mechanisms related to anxiety. However, it seems that this was not the case [30]. The most obvious explanation is the lack of changes in total exploration time, as we mentioned above. In addition, although there was an anxiogenic-like effect of 3.75 mg/kg PCA and HYP, there was no significant effect at the dose of 7.5 mg/kg.

As outlined above, antidepressants, mainly TCAs, widely used up to now, can clearly worsen cognitive function. The inquiry into whether HYP and/or PCA can improve cognitive impairment holds significant importance, especially given that TCAs generally hinder cognitive function through a mechanism reliant on cholinergic pathways. Therefore, we decided to investigate the efficacy of PCA and HYP at two doses—effective (7.5 mg/kg) and ineffective (1.875 mg/kg)—in antagonizing cholinergic hypofunction produced by treatment with scopolamine toward short-term and long-term memory. The reported research indicated object memory impairing effects of scopolamine (1 mg/kg) in either a 1- or 24-h delay in mice. There are findings that showed that the administration of scopolamine produced impairment in the NOR task, indicating the involvement of cholinergic neurotransmission in the regulation of this form of learning [33]. Despite the impact of scopolamine on all the examined groups, the animals exhibited heightened agitation, leading to increased locomotor activity. Nonetheless, the scopolamine-induced hyperactivity, evaluated through a specific motor activity task, solely represents a behavioral effect similar to that induced by numerous anticholinergic drugs, without affecting cognitive performance [34,35]. The rise in locomotor activity triggered by scopolamine does not seem to align with the findings presented, indicating a distinct scopolamine-induced memory decline. In reality, the total exploration time did not vary among the experimental groups, and scopolamine was administered just once, 15 min before commencing the acquisition trial. This study revealed that the dosage of 7.5 mg/kg of HYP successfully counteracted the effects of co-administered scopolamine in the model of long-term memory impairment induced by scopolamine. But, it should be noted that HYP at the same dose of 7.5 mg/kg did not prevent the scopolamine-induced deficits in short-term memory. The result is the subject of two different and speculative explanations. One consideration was the potential delayed activity of HYP metabolites. Secondly, we cannot dismiss the likelihood that the apparent failure of HYP to counteract scopolamine-induced short-term memory loss in the NOR task might be linked to the higher dose of scopolamine utilized in this particular setup [35]. PCA, administered at 7.5 mg/kg, was observed to prevent both short- and long-term memory impairment induced by scopolamine, highlighting the anti-amnestic properties of this polyphenol. Our findings align with earlier research by Krzysztoforska et al. [36], indicating that the chronic administration of PCA effectively restored serotonergic and dopaminergic neurotransmission and improved memory in a D-galactose (D-gal)-induced model of memory impairment in rats. Likewise, Krzysztoforska et al. [37] illustrated that repeated administration of PCA in rats enhanced memory consolidation and the retrieval of acquired information, effectively preventing adverse alterations induced by pyrithiamine-induced thiamine deficiency (PTD), a model mirroring Wernicke–Korsakoff syndrome.

The current preclinical and clinical data on body weight changes are ambiguous and dependent on the drug used. Upon reviewing published, controlled clinical trials, it has been observed that numerous depressed patients encounter weight gain while undergoing treatment with TCAs and certain newer antidepressants, such as mirtazapine, paroxetine, or venlafaxine [38]. On the other hand, some SSRIs, like fluoxetine or Ser, were found to inhibit weight gain and decrease food intake. In general, individuals using presently available antidepressants may face notable issues concerning body weight that can contribute to patient noncompliance and potentially result in medical complications [38,39]. Thus, in the present study, the body weight of mice was measured alongside chronic drug treatment. In both the PCA- and HYP-treated groups and the vehicle-treated group, body weights showed a similar increase over time. These results indicated that repeated treatment with polyphenols at the dose of 3.75 mg/kg did not affect body weight gain. On the contrary, only slight weight gain was observed following repeated administration of 15 mg/kg of Ser.

The primary theory explaining the pathophysiology of depression suggests that the condition arises because of disrupted monoaminergic transmission, which includes the serotonin system [40]. Numerous clinical and preclinical studies indicate that a disturbance in serotonergic activity may be associated with MDD. Although antidepressants acting through the enhancement of serotonergic neurotransmission have two primary limitations—low efficacy and slowness of action—they are the most commonly used group of drugs affecting the CNS [5]. It is known that most clinically useful antidepressant drugs potentiate, either directly or indirectly, the action of serotonin in the brain. To further understand the possible mechanism of PCA and HYP actions, the binding affinity at 5-HT_1A_ and 5-HT_2A_ receptors was assessed, and the peripheral serotonin level was measured. Our experiments revealed the compounds have no affinity for the above-mentioned receptors, whereas a significant increase in the serum serotonin level was observed after a prolonged administration of PCA and HYP at a dose of 3.75 mg/kg. This finding aligns with the hypothesis that prolonged receptor desensitization contributes to heightened firing rates of 5-HT neurons, which coincide with adaptive alterations in 5-HT receptors [3]. The relationship between synaptic 5-HT levels and peripheral 5-HT levels has piqued the curiosity of researchers. Numerous studies confirmed the possibility that the peripheral 5-HT level can reflect the 5-HT in synaptic cleft. Indeed, since the antidepressants’ suppression of platelet 5-HT uptake mirrors the inhibition of synaptic 5-HT uptake [41], it is rational to contemplate the potential use of peripheral serum, plasma, or platelet 5-HT as a biomarker for depression [42,43]. It is difficult to explain the lack of differences in the 5-HT level between experimental groups after acute treatment with PCA and HYP at a dose of 3.75 mg/kg. One intriguing potential explanation is that other monoamines besides 5-HT are involved in the antidepressant/anxiolytic/procognitive properties of the polyphenols. In our previous study, we discussed the interactions of PCA and HYP with different monoamines in the acute treatment. The pre-administration of *p*-chlorophenylalanine (PCPA 100 mg/kg, a serotonin synthesis inhibitor) effectively blocked the antidepressant-like effects of HYP (3.75 mg/kg) and fluoxetine (Flx, 15 mg/kg) but not PCA (3.75 mg/kg). Similarly, the synergistic effect was observed between the sub-effective dose of Flx (5 mg/kg) and HYP (0.94 mg/kg) but not between the sub-effective dose of Flx and PCA (0.94 mg/kg). Furthermore, the antidepressant-like effects of HYP and PCA were nullified by the administration of sulpiride (50 mg/kg), a D2 antagonist, and *α*-methyl-DL-tyrosine (AMPT 100 mg/kg, a catecholamine synthesis inhibitor). Sub-threshold doses of reboxetine (2 mg/kg) were able to enhance the effects of a sub-threshold dose of HYP (0.94 mg/kg) and PCA (0.94 mg/kg) in the FST [15]. It should also be emphasized that the tested polyphenols have completely different molecular skeletons than known antidepressants, such as SSRIs; Hence, their molecular mechanisms should be different. A lot of studies confirm that their antidepressant-like activity is related to their latent structure (for review, see [7,44]). And so, the tested hyperoside is a flavonol glycoside (a derivative of quercetin) with a molecular skeleton consisting of two aromatic rings along with an oxygen-containing heterocyclic benzopyran ring. It is known that flavonols like quercetin seem to affect the monoamine oxidase (MAO) activity. MAO is a key enzyme that is responsible for the metabolism of monoamines, i.e., NA, DA, and 5-HT. Based on the structure–activity relationship (SAR) study, it was concluded that the location of a hydroxy group(s) on the flavonoid A-ring impacted the potential antidepressant effects of flavonols, including HYP (for review, see [7]). Based on the current literature, as well as our research, we can conclude that the mechanism of the antidepressant action of HYP and PCA is rather indirect, and it does not depend on their direct effect on the receptors, e.g., 5-HT. It can be speculated that HYP and PCA can reverse monoamine neurotransmitter attenuations by blocking the reuptake of amines, blocking their metabolism, or influencing their synthesis. These speculations require further, more precise molecular studies.

The limitation of our study is the fact that antidepressant, anxiolytic, and procognitive/antiamnestic activities of the polyphenols were not tested using an animal model of mental disorder (e.g., an animal model of post-traumatic stress). Moreover, the memory-enhancing activity of PCA and HYP should be confirmed using other tests, such as passive avoidance or Morris water maze. It is worth noting that the presented data are preclinical studies. Therefore, clinical trials are needed to provide detailed information on the efficacy and safety of PCA and HYP for its CNS applications in humans.

## 4. Materials and Methods

### 4.1. Isolation of Hyperoside and Protocatechuic Acid

The procedure of isolation of protocatechuic acid (Figure 1a) and hyperoside (Figure 1b) was previously described [15].

### 4.2. Animals

The experiments involved adult-male Albino Swiss mice (25–27 g—the body weight on arrival from the Breeding Laboratory Animals of the Center for Experimental Medicine in Lublin, Poland), housed in cages containing 4–5 animals each at a room temperature of 22 ± 1 °C, with unrestricted access to food and water. During the chronic experiment, the body weight of mice was regularly monitored. All procedures were performed during the light phase, specifically between 9:00 a.m. and 3:00 p.m. Each experimental group comprised 8–10 animals. All behavioral experiments adhered to the European Community Council Directive for Care and Use of Laboratory Animals (2010/63/EU) and received approval from the Local Ethics Committee for Animal Experimentation (49/2016 and 134/2018).

Eight-to-ten–week-old male Cmdb:Wi rats (approx. 250 g) were used for tissue harvest. Rats were kept at the Central Laboratory of Experimental Animals, Medical University of Warsaw facilities. The animals were kept in standard environmental conditions, maintaining temperatures between 20 and 24 °C, with humidity levels at 55 ± 10%, following a 12/12 h light/dark cycle. Rats were group-housed in conventional T3H cages furnished with sawdust bedding and environmental enrichment. They were provided ad libitum access to fresh tap water and standard pelleted food.

### 4.3. Drugs

All substances were intraperitoneally (i.p.) administered at a consistent volume of 10 mL/kg body weight. HYP and PCA were dissolved in DMSO, reaching a final concentration of 0.1%. This solution was then suspended in 0.5% Tween-80 (1–2 drops) and subsequently diluted with saline solution (0.9% NaCl). Sertraline (Ser) hydrochloride (Cayman Chemical, Ann Arbor, MI, USA) and scopolamine (Sigma-Aldrich, St. Louis, MO, USA) were dissolved in 0.9% NaCl (saline). The control animals received the vehicle (0.9% NaCl). The dosages of the substances utilized were determined from prior research studies [15,45]. HYP, PCA, and Ser were given acutely 60 min before tests (EPM or novel object recognition test—NOR). Scopolamine was administered 15 min before the first trial of the NOR task.

### 4.4. Experimental Protocol for Chronic Experiment

**Scheme 1:**
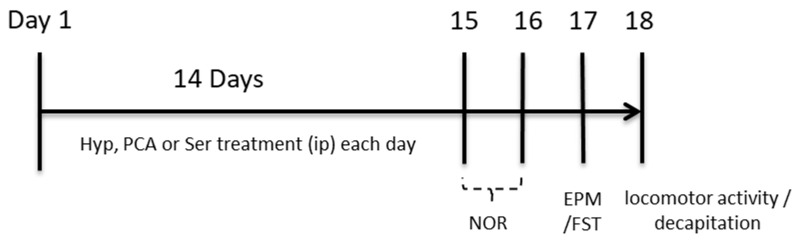
Scheme of experimental procedure for chronic experiment.

Mice were injected i.p. with saline, HYP or PCA (3.75 and 7.5 mg/kg), and Ser (15 mg/kg) once daily for 14 consecutive days. Following that period, the animals underwent behavioral tests in the subsequent sequence: one and two days (NOR), three days (EPM and forced swimming test—FST), and four days (a measurement of locomotor activity) after the last treatment (Scheme 1). Then, the mice were decapitated, which was followed by tissue collection and biochemical analysis.

### 4.5. Behavioral Tests

#### 4.5.1. Novel Object Recognition Test

The setup comprised a square open box crafted from Plexiglas^®^ (measuring 46 cm in length × 35 cm in height × 33 cm in width) and was illuminated by a lamp emitting light at an intensity of 10 lux, suspended 50 cm above the box. The objects used for discrimination—constructed from wood (prism), plastic (blue block), and glass (a small ball-shaped bottle)—were too weighty to be moved by the animals. The Novel Object Recognition (NOR) test was conducted following established protocols [29,46]. On the day preceding the test, each mouse spent 15 min in the empty box to acclimate to the environment. During the experimental days, the animals underwent three trials, with intervals of 1 h and 24 h between each trial. Each trial lasted 5 min. In the first trial (T1), the apparatus contained two identical objects (blue plastic blocks) positioned in two opposing corners, each located 10 cm away from the sidewall. A mouse was consistently positioned in the center of the box. Following T1, the mouse was returned to its home cage. Subsequently, after intervals of 1 h or 24 h, T2 and T3 were conducted. In T2 or T3, one of the objects from T1 was replaced with a new object (N), thereby presenting the mice with two objects: one familiar (F) and one new (N). To prevent olfactory cues, both the apparatus and the objects were cleaned after each mouse. Exploration was defined as directing the nose toward the object within a distance of no more than 2 cm and/or touching the object with the nose. Activities like turning around or sitting on the object were not considered exploratory behavior. The duration of exploration for each object during T1, T2, and T3 was manually recorded using a stopwatch. Discrimination between F and N during T2 or T3 was assessed by comparing the time spent exploring F with the time spent exploring N. Memory performance was evaluated using the discrimination index (DI), calculated for each animal using the formula: (N − F)/(N + F). This formula represents the difference in exploration time between N and F, adjusted against the total exploration time for both objects in T2 or T3. A higher discrimination index indicates stronger memory retention for familiar objects. The NOR test allowed for the evaluation of both short-term and long-term memory based on the duration between the pre-test and the subsequent retention test. Assessment of short-term memory occurred during the trial and was conducted 1 h after the training test. The latency time measured after 24 h provided insights into the assessment of long-term memory processes. In both instances, the drugs were administered prior to the pre-test, and their expected impact was to potentially disrupt the physiological acquisition of information [30,35].

#### 4.5.2. Elevated Plus-Maze (EPM) Test

The EPM experiments were conducted on mice following Lister’s method [18]. The EPM apparatus, constructed from plexiglass, comprised four arms arranged in a plus-sign configuration, elevated 38.5 cm above the floor, and illuminated by a subdued red light. Two arms were open (30 × 5 cm), while the other two were enclosed with dark walls (30 × 5 × 15 cm). These arms extended from a central platform measuring 5 × 5 cm. Each mouse was individually positioned at the central square of the plus-maze apparatus, facing the open arm, and observed for a duration of 5 min using a stopwatch. Post-evaluation, the equipment was cleaned with 70% ethanol and dried before the next mouse. For the analysis, open-arm activity was measured as the time spent on the open arms relative to the total time spent in both types of arms (open/total × 100) and the number of entries into open arms relative to the total number of entries into any arm (open/total × 100). A mouse was recorded as entering an open or closed arm when all four paws crossed over the dividing line.

#### 4.5.3. Spontaneous Locomotor Activity

The assessment of spontaneous locomotor activity was conducted using the Opto-Varimex-4 Auto-Track animal activity meter (Columbus Instruments, Columbus, OH, USA), following previously outlined procedures. The distance covered by the tested mouse (in cm) was recorded over 6 min and 60 min (during the acute scopolamine experiment) following a 5 min adaptation period. After each mouse was tested, the cages were cleaned using 70% ethanol [47].

#### 4.5.4. Forced Swimming Test

The experiment followed the procedure outlined by Porsolt et al. [20]. Each mouse was placed individually in a glass cylinder measuring 25 cm in height and 10 cm in diameter, filled with water at a constant temperature of 23–25 °C to a depth of 10 cm, and left undisturbed for 6 min. Immobility was defined as the mouse floating in the water and making minimal movements to keep its head above the surface. The duration of total immobility was recorded during the final 4 min of the 6 min test session.

### 4.6. Radioligand Receptor Binding

#### 4.6.1. Membrane Preparation

Sprague–Dawley rats were euthanized using an isoflurane overdose. Rats were decapitated; their brains were promptly removed and placed on ice. The hippocampi (for 5-HT_1A_ assays) and frontal cortices (for 5-HT_2A_ assays) were dissected on a Petri dish. Tissue from 10 rats was homogenized in 30 volumes of homogenization buffer (comprising 50 mM Tris-HCl, pH = 4.7, 1 mM EDTA, 1 mM dithiothreitol) using a hand-held Teflon-glass homogenizer. Subsequently, the homogenate was centrifuged at 48,000× *g* at 4 °C for 15 min. The resulting pellet was suspended, homogenized again in homogenization buffer, and incubated for 10 min at 36 °C. This centrifugation and suspension process was repeated twice. The final pellet was homogenized in 5 volumes of 50 mM Tris-HCl, pH = 7.4 buffer, and stored at −80 °C, not exceeding 6 months.

#### 4.6.2. Competitive 5-HT_1A_ and 5-HT_2A_ Binding Assays

In the 5-HT1A assay, 8–10 concentrations of each compound, equally spaced on a logarithmic scale, were incubated in duplicate with 1 nM [3H]8-OH-DPAT (specific activity: 200 Ci/mmol; Perkin Elmer, Waltham, MA, USA) for 60 min at 36 °C. This incubation took place in a 50 mM Tris-HCl (pH 7.4) buffer, supplemented with 0.1% ascorbate, 5 mM MgCl_2_, and 80µg of hippocampal membrane suspension.

For the 5-HT_2A_ assay, 160 µg of frontal cortex membrane suspension was incubated with 1 nM [3H]ketanserin (specific activity: 22.8 Ci/mmol; Perkin Elmer, Waltham, MA, USA) for 60 min at 36 °C. This incubation also occurred in a 50 mM Tris-HCl (pH 7.4) buffer, supplemented with 0.1% ascorbate and 3 mM CaCl_2_. Non-specific binding was determined using 10 μM serotonin in both assays. The final DMSO concentration in the assay was 5%.

Following incubation, the reaction mixture was applied onto Unifilter^®^ GF/C plates (Perkin Elmer, MA, USA) previously soaked in 0.4% PEI for 1 h using the FilterMate-96 Harvester (Perkin Elmer, Waltham, MA, USA). To separate bound ligands from free ones, each well was washed with 1.75 mL of 50 mM Tris-HCl (pH 7.4) buffer. Plates were air-dried overnight. When completely dry, the filters were saturated with 35 µL of Microscint-20 scintillation fluid (Perkin Elmer, Waltham, MA, USA) and left to equilibrate for 2 h. Radioactivity retained on the filters was counted in a MicroBeta^2^ LumiJet scintillation counter (Perkin Elmer, Waltham, MA, USA). Binding curves were fitted with a one-site non-linear regression model provided by GraphPad Prism 5.0 for Windows (GraphPad Software, Boston, MA, USA, www.graphpad.com—accessed on 30 November 2023). The Cheng–Prusoff equation was utilized to compute binding affinities (Ki) for each compound from two independent experiments.

### 4.7. Determination of Serotonin Level in the Serum of Mice

Mice were sacrificed immediately after the test by decapitation. Blood samples were collected from each animal in tubes without any anticoagulant and allowed to clot. Next, to obtain the serum, the blood samples were centrifuged at 1000× *g* for 10 min. Then, serum samples were collected in Eppendorf’s tubes and stored at −20 °C until used for the determination of serotonin level.

The concentration of serotonin was determined using a competitive enzyme-linked immunoassay (ELISA) kit designed for the accurate quantitative measurement of this biogenic amine in the serum, platelets, plasma, and urine (Serotonin ELISA Kit ab133053). All procedures were conducted in accordance with the manufacturer’s instructions (Abcam, Cambridge, UK).

### 4.8. Statistical Analysis

The results from in vivo experiments were calculated by two-way analysis of variance (ANOVA) (the analyses of the changes in body weight) and one-way ANOVA (other tests), followed by Bonferroni’s post hoc test, as appropriate. Radioligand binding data were statistically processed with the extra sum-of-squares F test. The results are presented as means ± standard errors of means (S.E.M). The level of *p* < 0.05 was considered statistically significant. All figures were prepared by GraphPad Prism version 5.00 for Windows (GraphPad Software, Boston, MA, USA, www.graphpad.com—accessed on 30 November 2023).

## 5. Conclusions

In summary, chronic administration of PCA and HYP exhibited antidepressant, anxiolytic, and cognitive-enhancing effects in mice without influencing their spontaneous locomotor activity or causing body weight gain. These behavioral outcomes align with the observed increase in serotonin levels in murine serum, supporting the involvement of the serotonergic system in the mechanisms of action of these polyphenols. Moreover, PCA and HYP demonstrated memory-enhancing properties and mitigated memory impairment induced by scopolamine following acute administration. Overall, these findings suggest that the polyphenols derived from *Impatiens glandulifera* hold the potential for treating mental disorders associated with cognitive dysfunction, warranting further comprehensive studies to delineate their pharmacological profile.

## Figures and Tables

**Table 1:** Binding affinities (Ki) of PCA, HYP, and reference compounds (5-HT and 8-OH-DPAT) at 5-HT_1A_ and 5-HT_2A_ receptors. Binding affinities of analyzed compounds were determined in the competitive radioligand binding assay using 5-HT_1A_ and 5-HT_2A_ selective probes–[^3^H]8-OH-DPAT and [^3^H]ketanserin, respectively. Ki values were calculated from the Cheng–Prusoff equation from two independent experiments. *** *p* < 0.001 HYP vs. 5-HT and ketanserin; ### *p* < 0.001 PCA vs. 5-HT and ketanserin.

	5-HT_1A_	5-HT_2A_	Selectivity (5-HT_2A_/5-HT_1A_)
Compound	µM (95% CI)	µM (95% CI)	
HYP	42.92 (25–74) ***	394 (255–608) ***	9.18
PCA	33.3 (17–63) ###	205 (106–390) ###	6.15
5-HT	0.007 (0.0045–0.011)	–	–
ketanserin	–	0.0023 (0.0013–0.041)	–

**Table 2:** Effects of PCA and HYP (1.875, 3.75, and 7.5 mg/kg, i.p.) acute and chronic administration on total exploration time of both objects in T2 displayed by different groups of mice during the short-term memory acquisition in NOR test. In the acute experiment, PCA and HYP (1.875, 3.75, and 7.5 mg/kg) were administered i.p. 60 min before the first trial (T1) of the NOR task. Total exploration time means the sum of the time of interest in both objects, measured 60 min after the training phase. In the chronic experiment, PCA and HYP (3.75 and 7.5 mg/kg) were administered i.p. for 14 consecutive days. Total exploration time was assessed 24 h after the last injection. The results are presented as mean ± SEM; ** *p* < 0.01 compared to the control group (Bonferroni’s test).

Compound (mg/kg)	Total Exploration Time in Seconds (Mean ± S.E.M.) During the Short-Term Memory Acquisition	
	Acute Treatment	Chronic Treatment
Saline	6.9 ± 0.61	27.71 ± 3.57
PCA 1.875	4.105 ± 1.076	-
3.75	7.73 ± 0.53	35.43 ± 3.682
7.5	10.31 ± 1.185	35.62 ± 2.52
HYP 1.875	5.2 ± 1.05	-
3.75	10.4 ± 1.56	35.05 ± 4.881
7.5	12.54 ± 1.378 **	25.69 ± 3.043

**Table 3:** Effects of PCA and HYP (1.875, 3.75, and 7.5 mg/kg, i.p.) acute and chronic administration on total exploration time of both objects in T3 displayed by different groups of mice during the long-term memory acquisition in NOR test. In the acute experiment, PCA and HYP (1.875, 3.75, and 7.5 mg/kg) were administered i.p. 60 min before the first trial (T1) of the NOR task. Total exploration time means the sum of the time of interest in both objects, measured 24 h after the training phase. In the chronic experiment, PCA and HYP (3.75 and 7.5 mg/kg) were administered i.p. for 14 consecutive days. Total exploration time was assessed 48 h after the last injection.

Compound (mg/kg)	Total Exploration Time in Seconds (Mean ± S.E.M.) During the Long-Term Memory Acquisition	
	Acute Treatment	Chronic Treatment
Saline	6.962 ± 0.49	26.93 ± 4.865
PCA 1.875	5.25 ± 1.08	-
3.75	8.835 ± 1.213	28.91 ± 4.488
7.5	9.844 ± 1.344	24.6 ± 3.383
HYP 1.875	5.67 ± 0.9	-
3.75	9.895 ± 1.866	27.01 ± 5.795
7.5	10.72 ± 1.141	24.08 ± 3.315

**Table 4:** Effects of PCA and HYP (1.875 and 7.5 mg/kg, ip) and/or scopolamine (1 mg/kg) acute administration on total exploration time of both objects in T2 and T3 displayed by different groups of mice during the short- or long-term memory acquisition in NOR test. HYP, PCA (1.875 and 7.5 mg/kg), or saline and/or scopolamine (1 mg/kg) were administered i.p. to mice 60 min and/or 15 min (for scopolamine) before the acquisition trial of NOR test. The compounds were injected combined with scopolamine.

Pretreatment	Treatment	Total Exploration Time in Seconds (Mean ± S.E.M.) During	
		Short-Term Memory Acquisition	Long-Term Memory Acquisition
Saline	Saline	8.127 ± 0.57	35.18 ± 2.186
Saline	Scopolamine 1 mg/kg	12.02 ± 1.6	43.6 ± 4.806
PCA 1.875 mg/kg	Scopolamine 1 mg/kg	7.02 ± 0.85	35.56 ± 2.341
7.5 mg/kg	Scopolamine 1 mg/kg	12.09 ± 0.93	43.85 ± 4.247
HYP 1.875 mg/kg	Scopolamine 1 mg/kg	8.18 ± 0.64	51.32 ± 3.156
7.5 mg/kg	Scopolamine 1 mg/kg	11.51 ± 1.6	37.19 ± 3.787

**Table 5:** Effects of PCA, HYP (3.75 and 7.5 mg/kg), and Ser (1 mg/kg) chronic (14 days) administration on the locomotor activity of mice. On the 18th day of the experiment, which was two days after the final administration of the compounds (as shown in Scheme 1), the locomotor activity of each animal was individually recorded in activity cages. Control group received 0.9% NaCl (saline). The data are presented as mean ± SEM of total distance traveled in centimeters within 6 min.

Treatment	Dose (mg/kg)	Mean of Distance Traveled ± S.E.M (cm) Within 6 min
Control (saline)	-	2324 ± 91.83
Sertraline	15	2217 ± 99.87
PCA	3.75 7.5	2368 ± 214.8 2152 ± 187.5
HYP	3.75 7.5	2553 ± 192.2 2730 ± 149.6

**Table 6:** Effect of PCA, HYP, and/or scopolamine acute administration on the locomotor activity of mice. The locomotor activity was recorded individually for each animal in activity cages within 6 min and 60 min. PCA and HYP (1.875 and 7.5 mg/kg) were injected i.p. 60 min prior to the test or 45 min prior to the administration of scopolamine (1 mg/kg), whereas scopolamine was injected i.p. 15 min prior to the test. The data are expressed as mean ± SEM of total distance traveled in centimeters. ** *p* < 0.01, *** *p* < 0.001 vs. saline + saline group (one-way ANOVA followed by Bonferroni’s test).

Pretreatment	Treatment	Mean of Distance Traveled ± S.E.M (cm) Within 6 min	Mean of Distance Traveled ± S.E.M (cm) Within 60 min
Saline	saline	580.3 ± 30	3450 ± 308.3
PCA 1.875 mg/kg	saline	492.1 ± 75.87	2127 ± 268.3
7.5 mg/kg	saline	565.7 ± 39.14	2204 ± 327.9
HYP 1.875 mg/kg	saline	562.3 ± 26.01	2423 ± 389.6
7.5 mg/kg	saline	546.7 ± 48.16	2181 ± 444.4
Saline	Scopolamine 1 mg/kg	985.3 ± 104 ***	6188 ± 381 **
PCA 1.875 mg/kg	Scopolamine 1 mg/kg	1028 ± 39.82	5162 ± 772.1
7.5 mg/kg	Scopolamine 1 mg/kg	915.6 ± 81.33	5741 ± 820.2
HYP 1.875 mg/kg	Scopolamine 1 mg/kg	1017 ± 68.36	6517 ± 560.2
7.5 mg/kg	Scopolamine 1 mg/kg	1021 ± 23.77	5428 ± 847.7

## Data Availability

Data are contained within the article.
